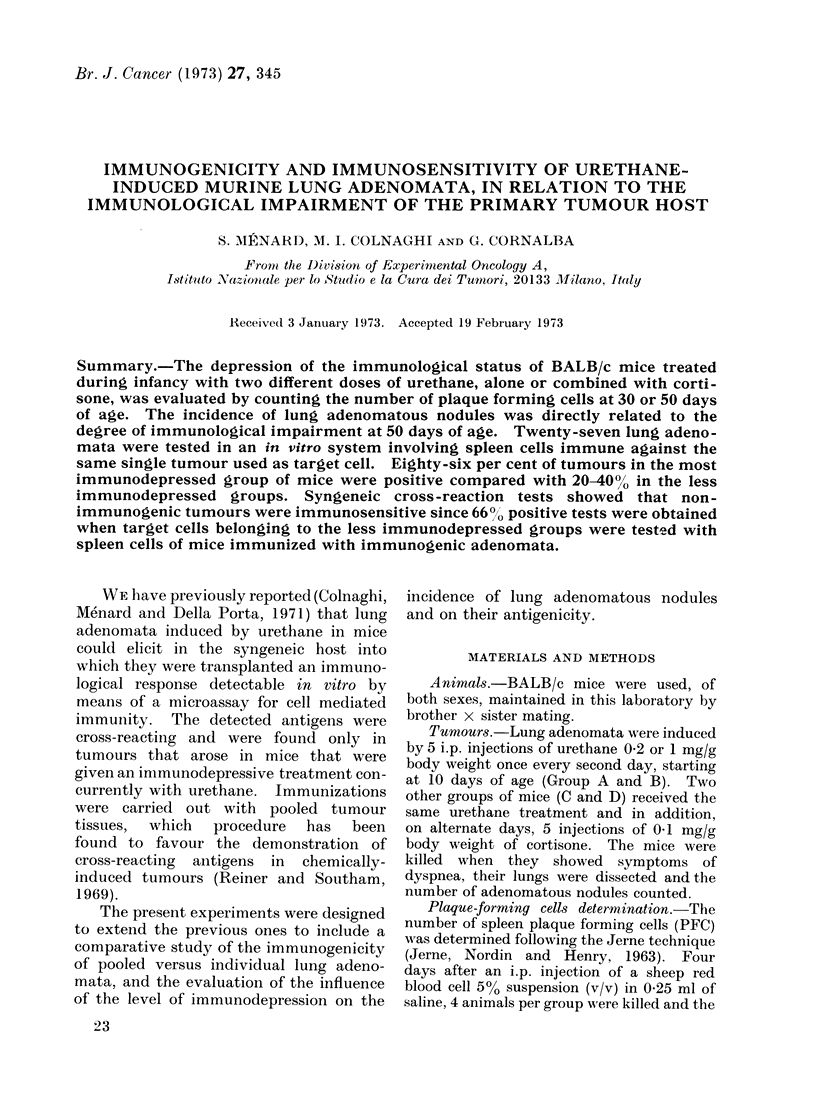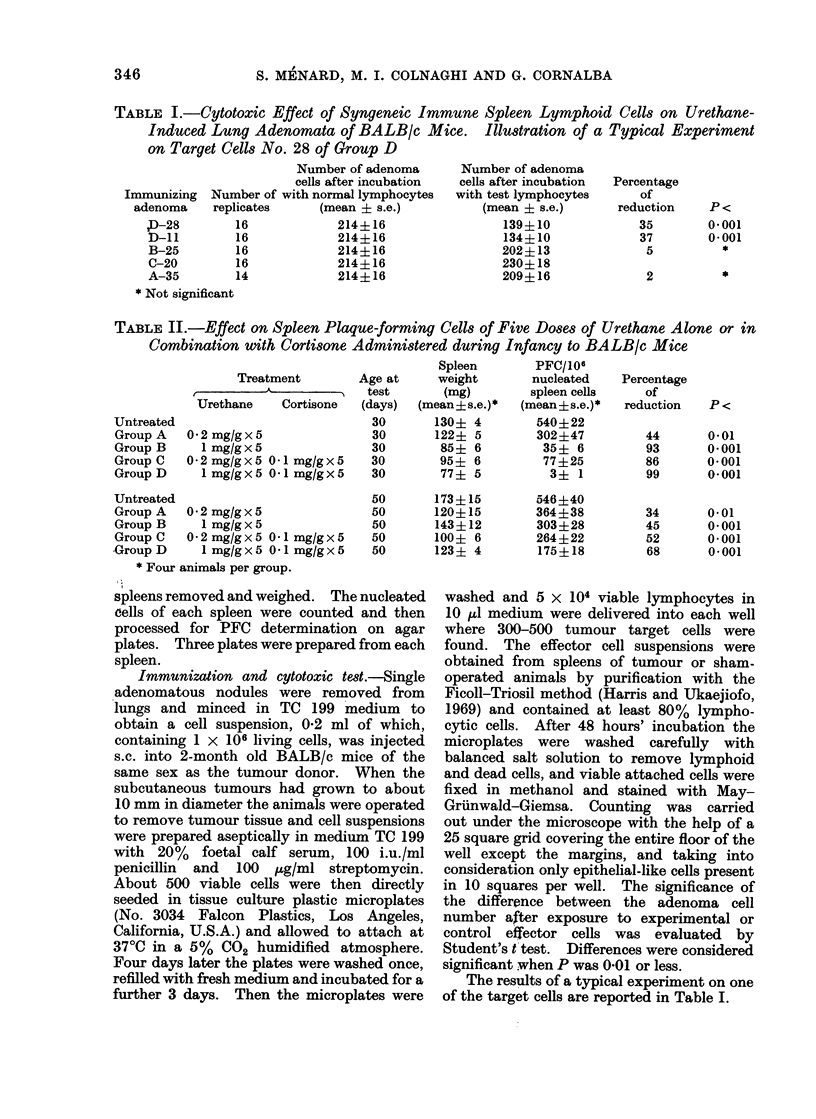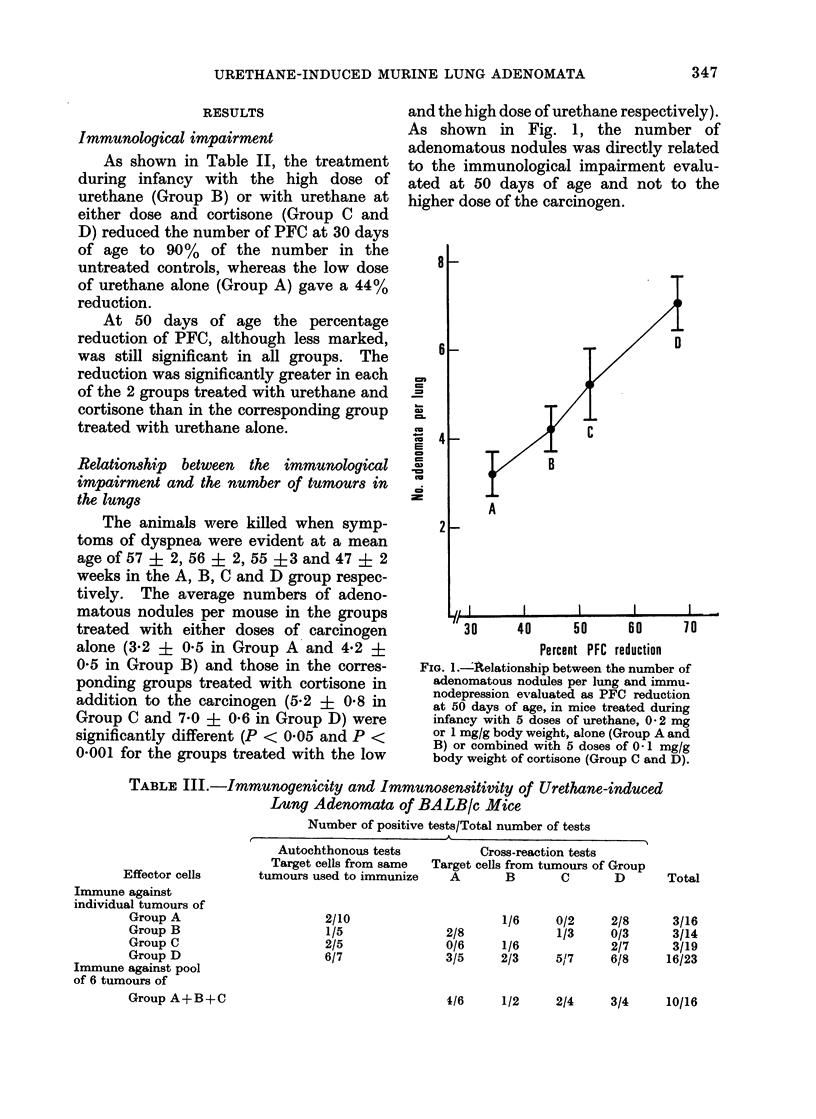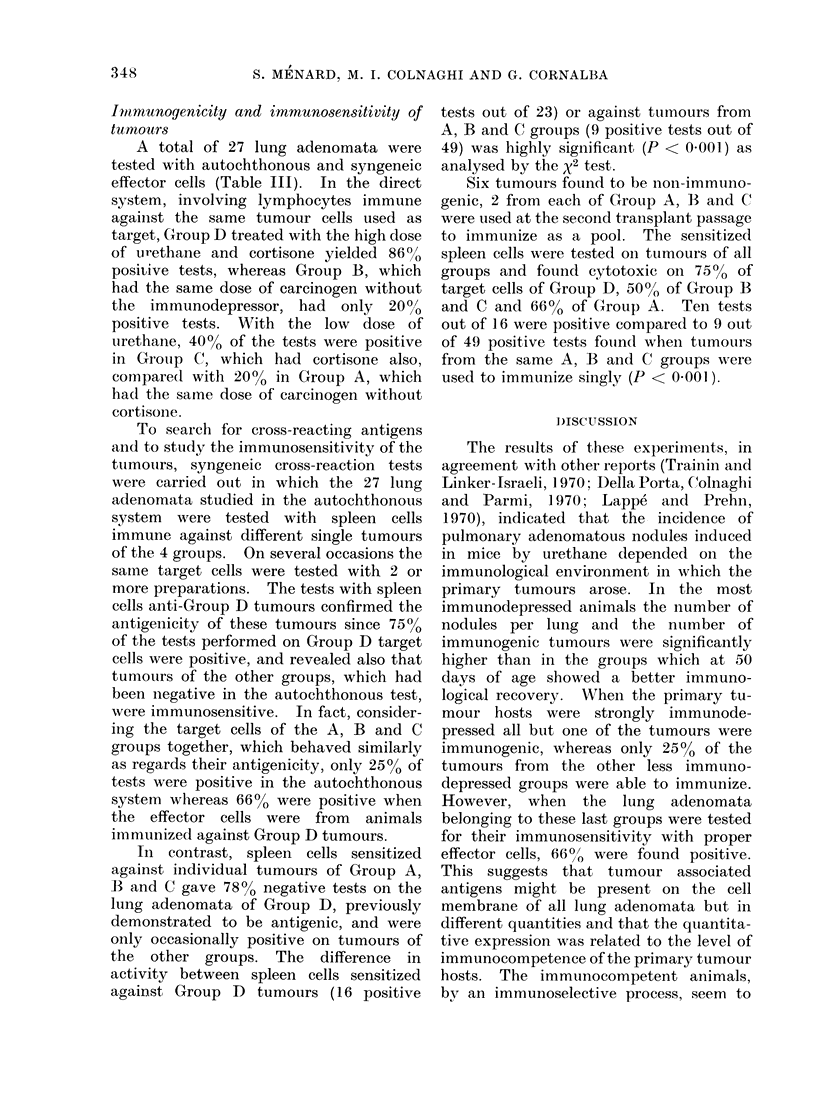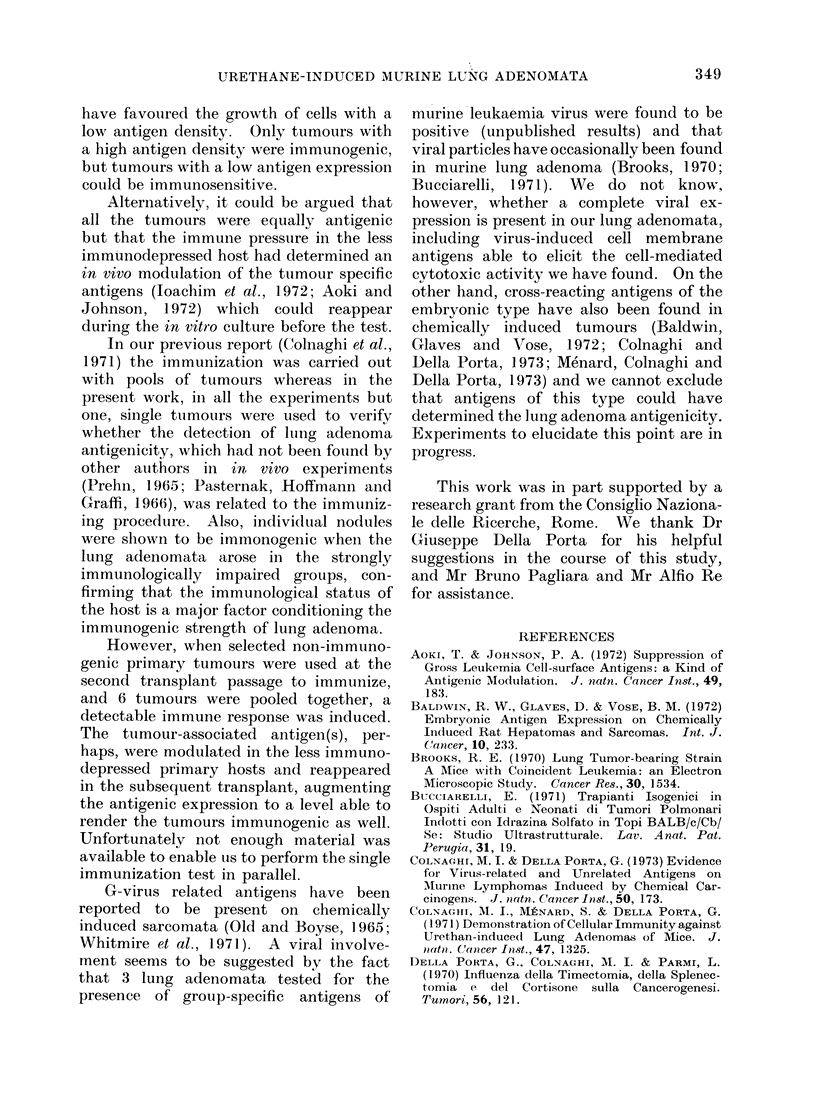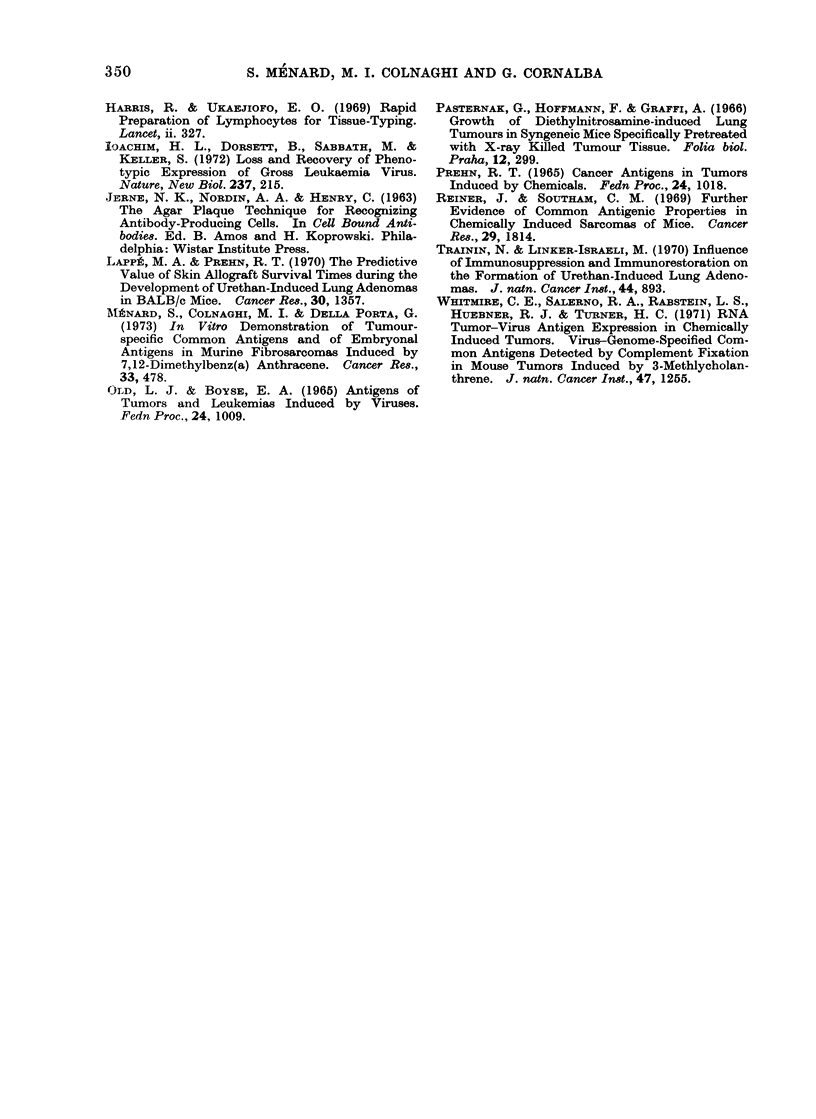# Immunogenicity and Immunosensitivity of Urethane-induced Murine Lung Adenomata, in Relation to the Immunological Impairment of the Primary Tumour Host

**DOI:** 10.1038/bjc.1973.42

**Published:** 1973-05

**Authors:** S. Ménard, M. I. Colnaghi, G. Cornalba

## Abstract

The depression of the immunological status of BALB/c mice treated during infancy with two different doses of urethane, alone or combined with cortisone, was evaluated by counting the number of plaque forming cells at 30 or 50 days of age. The incidence of lung adenomatous nodules was directly related to the degree of immunological impairment at 50 days of age. Twenty-seven lung adenomata were tested in an *in vitro* system involving spleen cells immune against the same single tumour used as target cell. Eighty-six per cent of tumours in the most immunodepressed group of mice were positive compared with 20-40% in the less immunodepressed groups. Syngeneic cross-reaction tests showed that non-immunogenic tumours were immunosensitive since 66% positive tests were obtained when target cells belonging to the less immunodepressed groups were tested with spleen cells of mice immunized with immunogenic adenomata.


*Br. J. Cancer* (1973) **27**, 345

### IMMUNOGENICITY AND IMMUNOSENSITIVITY OF URETHANE-INDUCED MURINE LUNG ADENOMATA, IN RELATION TO THE IMMUNOLOGICAL IMPAIRMENT OF THE PRIMARY TUMOUR HOST

S. MÉNARD, M. I. COLNAGHI AND G. CORNALBA

*From the Division of Experimental Oncology A,*

*Istituto Nazionale per lo Studio e la Cura dei Tumori,* 20133 *Milano, Italy*

Received 3 January 1973. Accepted 19 February 1973

**Summary.—The depression of the immunological status of BALB/c mice treated during infancy with two different doses of urethane, alone or combined with cortisone, was evaluated by counting the number of plaque forming cells at 30 or 50 days of age. The incidence of lung adenomatous nodules was directly related to the degree of immunological impairment at 50 days of age. Twenty-seven lung adenomata were tested in an *in vitro* system involving spleen cells immune against the same single tumour used as target cell. Eighty-six per cent of tumours in the most immunodepressed group of mice were positive compared with 20–40% in the less immunodepressed groups. Syngeneic cross-reaction tests showed that non-immunogenic tumours were immunosensitive since 66% positive tests were obtained when target cells belonging to the less immunodepressed groups were tested with spleen cells of mice immunized with immunogenic adenomata.**

WE have previously reported (Colnaghi, Ménard and Della Porta, 1971) that lung adenomata induced by urethane in mice could elicit in the syngeneic host into which they were transplanted an immunological response detectable *in vitro* by means of a microassay for cell mediated immunity. The detected antigens were cross-reacting and were found only in tumours that arose in mice that were given an immunodepressive treatment concurrently with urethane. Immunizations were carried out with pooled tumour tissues, which procedure has been found to favour the demonstration of cross-reacting antigens in chemically-induced tumours (Reiner and Southam, 1969).

The present experiments were designed to extend the previous ones to include a comparative study of the immunogenicity of pooled versus individual lung adenomata, and the evaluation of the influence of the level of immunodepression on the incidence of lung adenomatous nodules and on their antigenicity.

### MATERIALS AND METHODS

*Animals.*—BALB/c mice were used, of both sexes, maintained in this laboratory by brother × sister mating.

*Tumours.*—Lung adenomata were induced by 5 i.p. injections of urethane 0·2 or 1 mg/g body weight once every second day, starting at 10 days of age (Group A and B). Two other groups of mice (C and D) received the same urethane treatment and in addition, on alternate days, 5 injections of 0·1 mg/g body weight of cortisone. The mice were killed when they showed symptoms of dyspnea, their lungs were dissected and the number of adenomatous nodules counted.

*Plaque-forming cells determination.*—The number of spleen plaque forming cells (PFC) was determined following the Jerne technique (Jerne, Nordin and Henry, 1963). Four days after an i.p. injection of a sheep red blood cell 5% suspension (v/v) in 0·25 ml of saline, 4 animals per group were killed and the

TABLE I.—*Cytotoxic Effect of Syngeneic Immune Spleen Lymphoid Cells on Urethane-Induced Lung Adenomata of BALB/c Mice. Illustration of a Typical Experiment on Target Cells No. 28 of Group D*

| Immunizing adenoma | Number of replicates | Number of adenoma cells after incubation with normal lymphocytes (mean ± s.e.) | Number of adenoma cells after incubation with test lymphocytes (mean ± s.e.) | Percentage of reduction | $P<$ |
|---|---|---|---|---|---|
| D-28 | 16 | 214±16 | 139±10 | 35 | 0·001 |
| D-11 | 16 | 214±16 | 134±10 | 37 | 0·001 |
| B-25 | 16 | 214±16 | 202±13 | 5 | * |
| C-20 | 16 | 214±16 | 230±18 | | |
| A-35 | 14 | 214±16 | 209±16 | 2 | * |

* Not significant

TABLE II.—*Effect on Spleen Plaque-forming Cells of Five Doses of Urethane Alone or in Combination with Cortisone Administered during Infancy to BALB/c Mice*

| | Treatment | | Age at test (days) | Spleen weight (mg) (mean±s.e.)* | PFC/$10^6$ nucleated spleen cells (mean±s.e.)* | Percentage of reduction | $P<$ |
|---|---|---|---|---|---|---|---|
| | Urethane | Cortisone | | | | | |
| Untreated | | | 30 | 130± 4 | 540±22 | | |
| Group A | 0·2 mg/g×5 | | 30 | 122± 5 | 302±47 | 44 | 0·01 |
| Group B | 1 mg/g×5 | | 30 | 85± 6 | 35± 6 | 93 | 0·001 |
| Group C | 0·2 mg/g×5 | 0·1 mg/g×5 | 30 | 95± 6 | 77±25 | 86 | 0·001 |
| Group D | 1 mg/g×5 | 0·1 mg/g×5 | 30 | 77± 5 | 3± 1 | 99 | 0·001 |
| Untreated | | | 50 | 173±15 | 546±40 | | |
| Group A | 0·2 mg/g×5 | | 50 | 120±15 | 364±38 | 34 | 0·01 |
| Group B | 1 mg/g×5 | | 50 | 143±12 | 303±28 | 45 | 0·001 |
| Group C | 0·2 mg/g×5 | 0·1 mg/g×5 | 50 | 100± 6 | 264±22 | 52 | 0·001 |
| Group D | 1 mg/g×5 | 0·1 mg/g×5 | 50 | 123± 4 | 175±18 | 68 | 0·001 |

* Four animals per group.

spleens removed and weighed. The nucleated cells of each spleen were counted and then processed for PFC determination on agar plates. Three plates were prepared from each spleen.

*Immunization and cytotoxic test.*—Single adenomatous nodules were removed from lungs and minced in TC 199 medium to obtain a cell suspension, 0·2 ml of which, containing 1 × $10^6$ living cells, was injected s.c. into 2-month old BALB/c mice of the same sex as the tumour donor. When the subcutaneous tumours had grown to about 10 mm in diameter the animals were operated to remove tumour tissue and cell suspensions were prepared aseptically in medium TC 199 with 20% foetal calf serum, 100 i.u./ml penicillin and 100 μg/ml streptomycin. About 500 viable cells were then directly seeded in tissue culture plastic microplates (No. 3034 Falcon Plastics, Los Angeles, California, U.S.A.) and allowed to attach at 37°C in a 5% $CO_2$ humidified atmosphere. Four days later the plates were washed once, refilled with fresh medium and incubated for a further 3 days. Then the microplates were washed and 5 × $10^4$ viable lymphocytes in 10 μl medium were delivered into each well where 300–500 tumour target cells were found. The effector cell suspensions were obtained from spleens of tumour or sham-operated animals by purification with the Ficoll–Triosil method (Harris and Ukaejiofo, 1969) and contained at least 80% lymphocytic cells. After 48 hours' incubation the microplates were washed carefully with balanced salt solution to remove lymphoid and dead cells, and viable attached cells were fixed in methanol and stained with May–Grünwald–Giemsa. Counting was carried out under the microscope with the help of a 25 square grid covering the entire floor of the well except the margins, and taking into consideration only epithelial-like cells present in 10 squares per well. The significance of the difference between the adenoma cell number after exposure to experimental or control effector cells was evaluated by Student's $t$ test. Differences were considered significant when $P$ was 0·01 or less.

The results of a typical experiment on one of the target cells are reported in Table I.

RESULTS

*Immunological impairment*

As shown in Table II, the treatment during infancy with the high dose of urethane (Group B) or with urethane at either dose and cortisone (Group C and D) reduced the number of PFC at 30 days of age to 90% of the number in the untreated controls, whereas the low dose of urethane alone (Group A) gave a 44% reduction.

At 50 days of age the percentage reduction of PFC, although less marked, was still significant in all groups. The reduction was significantly greater in each of the 2 groups treated with urethane and cortisone than in the corresponding group treated with urethane alone.

*Relationship between the immunological impairment and the number of tumours in the lungs*

The animals were killed when symptoms of dyspnea were evident at a mean age of 57 ± 2, 56 ± 2, 55 ±3 and 47 ± 2 weeks in the A, B, C and D group respectively. The average numbers of adenomatous nodules per mouse in the groups treated with either doses of carcinogen alone (3·2 ± 0·5 in Group A and 4·2 ± 0·5 in Group B) and those in the corresponding groups treated with cortisone in addition to the carcinogen (5·2 ± 0·8 in Group C and 7·0 ± 0·6 in Group D) were significantly different ($P < 0{\cdot}05$ and $P < 0{\cdot}001$ for the groups treated with the low and the high dose of urethane respectively). As shown in Fig. 1, the number of adenomatous nodules was directly related to the immunological impairment evaluated at 50 days of age and not to the higher dose of the carcinogen.

FIG. 1.—Relationship between the number of adenomatous nodules per lung and immunodepression evaluated as PFC reduction at 50 days of age, in mice treated during infancy with 5 doses of urethane, 0·2 mg or 1 mg/g body weight, alone (Group A and B) or combined with 5 doses of 0·1 mg/g body weight of cortisone (Group C and D).

TABLE III.—*Immunogenicity and Immunosensitivity of Urethane-induced Lung Adenomata of BALB/c Mice*

| Effector cells | Number of positive tests/Total number of tests: Autochthonous tests — Target cells from same tumours used to immunize | Cross-reaction tests — Target cells from tumours of Group A | B | C | D | Total |
|---|---|---|---|---|---|---|
| Immune against individual tumours of | | | | | | |
| Group A | 2/10 | | 1/6 | 0/2 | 2/8 | 3/16 |
| Group B | 1/5 | 2/8 | | 1/3 | 0/3 | 3/14 |
| Group C | 2/5 | 0/6 | 1/6 | | 2/7 | 3/19 |
| Group D | 6/7 | 3/5 | 2/3 | 5/7 | 6/8 | 16/23 |
| Immune against pool of 6 tumours of | | | | | | |
| Group A+B+C | | 4/6 | 1/2 | 2/4 | 3/4 | 10/16 |

*Immunogenicity and immunosensitivity of tumours*

A total of 27 lung adenomata were tested with autochthonous and syngeneic effector cells (Table III). In the direct system, involving lymphocytes immune against the same tumour cells used as target, Group D treated with the high dose of urethane and cortisone yielded 86% positive tests, whereas Group B, which had the same dose of carcinogen without the immunodepressor, had only 20% positive tests. With the low dose of urethane, 40% of the tests were positive in Group C, which had cortisone also, compared with 20% in Group A, which had the same dose of carcinogen without cortisone.

To search for cross-reacting antigens and to study the immunosensitivity of the tumours, syngeneic cross-reaction tests were carried out in which the 27 lung adenomata studied in the autochthonous system were tested with spleen cells immune against different single tumours of the 4 groups. On several occasions the same target cells were tested with 2 or more preparations. The tests with spleen cells anti-Group D tumours confirmed the antigenicity of these tumours since 75% of the tests performed on Group D target cells were positive, and revealed also that tumours of the other groups, which had been negative in the autochthonous test, were immunosensitive. In fact, considering the target cells of the A, B and C groups together, which behaved similarly as regards their antigenicity, only 25% of tests were positive in the autochthonous system whereas 66% were positive when the effector cells were from animals immunized against Group D tumours.

In contrast, spleen cells sensitized against individual tumours of Group A, B and C gave 78% negative tests on the lung adenomata of Group D, previously demonstrated to be antigenic, and were only occasionally positive on tumours of the other groups. The difference in activity between spleen cells sensitized against Group D tumours (16 positive tests out of 23) or against tumours from A, B and C groups (9 positive tests out of 49) was highly significant ($P < 0{\cdot}001$) as analysed by the $\chi^2$ test.

Six tumours found to be non-immunogenic, 2 from each of Group A, B and C were used at the second transplant passage to immunize as a pool. The sensitized spleen cells were tested on tumours of all groups and found cytotoxic on 75% of target cells of Group D, 50% of Group B and C and 66% of Group A. Ten tests out of 16 were positive compared to 9 out of 49 positive tests found when tumours from the same A, B and C groups were used to immunize singly ($P < 0{\cdot}001$).

### DISCUSSION

The results of these experiments, in agreement with other reports (Trainin and Linker-Israeli, 1970; Della Porta, Colnaghi and Parmi, 1970; Lappé and Prehn, 1970), indicated that the incidence of pulmonary adenomatous nodules induced in mice by urethane depended on the immunological environment in which the primary tumours arose. In the most immunodepressed animals the number of nodules per lung and the number of immunogenic tumours were significantly higher than in the groups which at 50 days of age showed a better immunological recovery. When the primary tumour hosts were strongly immunodepressed all but one of the tumours were immunogenic, whereas only 25% of the tumours from the other less immunodepressed groups were able to immunize. However, when the lung adenomata belonging to these last groups were tested for their immunosensitivity with proper effector cells, 66% were found positive. This suggests that tumour associated antigens might be present on the cell membrane of all lung adenomata but in different quantities and that the quantitative expression was related to the level of immunocompetence of the primary tumour hosts. The immunocompetent animals, by an immunoselective process, seem to have favoured the growth of cells with a low antigen density. Only tumours with a high antigen density were immunogenic, but tumours with a low antigen expression could be immunosensitive.

Alternatively, it could be argued that all the tumours were equally antigenic but that the immune pressure in the less immunodepressed host had determined an *in vivo* modulation of the tumour specific antigens (Ioachim *et al.*, 1972; Aoki and Johnson, 1972) which could reappear during the *in vitro* culture before the test.

In our previous report (Colnaghi *et al.*, 1971) the immunization was carried out with pools of tumours whereas in the present work, in all the experiments but one, single tumours were used to verify whether the detection of lung adenoma antigenicity, which had not been found by other authors in *in vivo* experiments (Prehn, 1965; Pasternak, Hoffmann and Graffi, 1966), was related to the immunizing procedure. Also, individual nodules were shown to be immonogenic when the lung adenomata arose in the strongly immunologically impaired groups, confirming that the immunological status of the host is a major factor conditioning the immunogenic strength of lung adenoma.

However, when selected non-immunogenic primary tumours were used at the second transplant passage to immunize, and 6 tumours were pooled together, a detectable immune response was induced. The tumour-associated antigen(s), perhaps, were modulated in the less immunodepressed primary hosts and reappeared in the subsequent transplant, augmenting the antigenic expression to a level able to render the tumours immunogenic as well. Unfortunately not enough material was available to enable us to perform the single immunization test in parallel.

G-virus related antigens have been reported to be present on chemically induced sarcomata (Old and Boyse, 1965; Whitmire *et al.*, 1971). A viral involvement seems to be suggested by the fact that 3 lung adenomata tested for the presence of group-specific antigens of murine leukaemia virus were found to be positive (unpublished results) and that viral particles have occasionally been found in murine lung adenoma (Brooks, 1970; Bucciarelli, 1971). We do not know, however, whether a complete viral expression is present in our lung adenomata, including virus-induced cell membrane antigens able to elicit the cell-mediated cytotoxic activity we have found. On the other hand, cross-reacting antigens of the embryonic type have also been found in chemically induced tumours (Baldwin, Glaves and Vose, 1972; Colnaghi and Della Porta, 1973; Ménard, Colnaghi and Della Porta, 1973) and we cannot exclude that antigens of this type could have determined the lung adenoma antigenicity. Experiments to elucidate this point are in progress.

This work was in part supported by a research grant from the Consiglio Nazionale delle Ricerche, Rome. We thank Dr Giuseppe Della Porta for his helpful suggestions in the course of this study, and Mr Bruno Pagliara and Mr Alfio Re for assistance.

### REFERENCES

AOKI, T. & JOHNSON, P. A. (1972) Suppression of Gross Leukemia Cell-surface Antigens: a Kind of Antigenic Modulation. *J. natn. Cancer Inst.*, **49,** 183.

BALDWIN, R. W., GLAVES, D. & VOSE, B. M. (1972) Embryonic Antigen Expression on Chemically Induced Rat Hepatomas and Sarcomas. *Int. J. Cancer*, **10**, 233.

BROOKS, R. E. (1970) Lung Tumor-bearing Strain A Mice with Coincident Leukemia: an Electron Microscopic Study. *Cancer Res.*, **30**, 1534.

BUCCIARELLI, E. (1971) Trapianti Isogenici in Ospiti Adulti e Neonati di Tumori Polmonari Indotti con Idrazina Solfato in Topi BALB/c/Cb/Se: Studio Ultrastrutturale. *Lav. Anat. Pat. Perugia*, **31**, 19.

COLNAGHI, M. I. & DELLA PORTA, G. (1973) Evidence for Virus-related and Unrelated Antigens on Murine Lymphomas Induced by Chemical Carcinogens. *J. natn. Cancer Inst.*, **50**, 173.

COLNAGHI, M. I., MÉNARD, S. & DELLA PORTA, G. (1971) Demonstration of Cellular Immunity against Urethan-induced Lung Adenomas of Mice. *J. natn. Cancer Inst.*, **47**, 1325.

DELLA PORTA, G., COLNAGHI, M. I. & PARMI, L. (1970) Influenza della Timectomia, della Splenectomia e del Cortisone sulla Cancerogenesi. *Tumori*, **56**, 121.

HARRIS, R. & UKAEJIOFO, E. O. (1969) Rapid Preparation of Lymphocytes for Tissue-Typing. *Lancet*, ii. 327.

IOACHIM, H. L., DORSETT, B., SABBATH, M. & KELLER, S. (1972) Loss and Recovery of Phenotypic Expression of Gross Leukaemia Virus. *Nature, New Biol.* **237**, 215.

JERNE, N. K., NORDIN, A. A. & HENRY, C. (1963) The Agar Plaque Technique for Recognizing Antibody-Producing Cells. In *Cell Bound Antibodies*. Ed. B. Amos and H. Koprowski. Philadelphia: Wistar Institute Press.

LAPPÉ, M. A. & PREHN, R. T. (1970) The Predictive Value of Skin Allograft Survival Times during the Development of Urethan-Induced Lung Adenomas in BALB/c Mice. *Cancer Res.*, **30**, 1357.

MÉNARD, S., COLNAGHI, M. I. & DELLA PORTA, G. (1973) *In Vitro* Demonstration of Tumour-specific Common Antigens and of Embryonal Antigens in Murine Fibrosarcomas Induced by 7,12-Dimethylbenz(a) Anthracene. *Cancer Res.*, **33**, 478.

OLD, L. J. & BOYSE, E. A. (1965) Antigens of Tumors and Leukemias Induced by Viruses. *Fedn Proc.*, **24**, 1009.

PASTERNAK, G., HOFFMANN, F. & GRAFFI, A. (1966) Growth of Diethylnitrosamine-induced Lung Tumours in Syngeneic Mice Specifically Pretreated with X-ray Killed Tumour Tissue. *Folia biol. Praha*, **12**, 299.

PREHN, R. T. (1965) Cancer Antigens in Tumors Induced by Chemicals. *Fedn Proc.*, **24**, 1018.

REINER, J. & SOUTHAM, C. M. (1969) Further Evidence of Common Antigenic Properties in Chemically Induced Sarcomas of Mice. *Cancer Res.*, **29**, 1814.

TRAININ, N. & LINKER-ISRAELI, M. (1970) Influence of Immunosuppression and Immunorestoration on the Formation of Urethan-Induced Lung Adenomas. *J. natn. Cancer Inst.*, **44**, 893.

WHITMIRE, C. E., SALERNO, R. A., RABSTEIN, L. S., HUEBNER, R. J. & TURNER, H. C. (1971) RNA Tumor–Virus Antigen Expression in Chemically Induced Tumors. Virus–Genome-Specified Common Antigens Detected by Complement Fixation in Mouse Tumors Induced by 3-Methlycholanthrene. *J. natn. Cancer Inst.*, **47**, 1255.